# Harnessing Healing Power: A Comprehensive Review on Platelet-Rich Plasma in Compound Fracture Care

**DOI:** 10.7759/cureus.52722

**Published:** 2024-01-22

**Authors:** Prathamesh Kale, Sandeep Shrivastava, Aditya Pundkar, Prashanth Balusani

**Affiliations:** 1 Orthopaedic Surgery, Jawaharlal Nehru Medical College, Datta Meghe Institute of Medical Sciences, Wardha, IND; 2 Orthopaedics, Jawaharlal Nehru Medical College, Datta Meghe Institute of Medical Sciences, Wardha, IND; 3 Orthopaedics and Traumatology, Jawaharlal Nehru Medical College, Datta Meghe Institute of Medical Sciences, Wardha, IND

**Keywords:** ethical considerations, inflammation reduction, soft tissue repair, bone healing, compound fractures, platelet-rich plasma (prp)

## Abstract

This comprehensive review explores the applications of platelet-rich plasma (PRP) in the context of compound fracture care, providing a thorough examination of its biological mechanisms, preparation techniques, and clinical implications. The analysis highlights PRP's potential in accelerating bone healing, enhancing soft tissue repair, reducing inflammation and infection risks, and managing pain during fracture recovery. The review underscores the importance of ethical and regulatory considerations in integrating PRP into orthopaedic practice, emphasising informed consent, transparent patient communication, and ongoing monitoring of ethical concerns. Looking ahead, the implications for the future of compound fracture care suggest a transformative shift with the potential for personalised medicine approaches and emerging technologies. However, the conclusion calls for a balanced perspective, acknowledging the promising applications of PRP while emphasising the need for responsible and ethical use. The collaborative efforts of healthcare professionals, researchers, and regulatory bodies are crucial in navigating this evolving landscape and harnessing the healing power of PRP to redefine orthopaedic care for individuals with compound fractures.

## Introduction and background

Platelet-rich plasma (PRP) is a regenerative medicine approach that has garnered significant attention recently for its potential to enhance tissue healing and regeneration. PRP is derived from the patient's blood and contains a concentrated form of platelets, growth factors, and other bioactive proteins. Platelets play a crucial role in the body's natural healing processes, and PRP harnesses this innate healing power to accelerate and optimise the recovery of injured tissues [1]. The challenges in achieving efficient healing are pronounced in compound fractures, where bones are broken and protrude through the skin. Traditional treatments for compound fractures often involve surgical intervention, immobilisation, and a lengthy rehabilitation process. Exploring alternative therapies, such as PRP, presents a promising avenue for improving outcomes in compound fracture care [2].

Compound fractures pose a unique set of challenges compared to closed fractures. The risk of infection, delayed healing, and complications related to soft tissue damage are heightened in cases of compound fractures [3]. Efficient and robust healing is crucial to minimise the risk of long-term complications, including chronic pain, impaired mobility, and the development of secondary conditions. Addressing the specific needs of compound fractures is essential to optimise patient outcomes and improve the overall quality of fracture care [4].

This comprehensive review aims to delve into the multifaceted aspects of PRP therapy in the context of compound fractures. By exploring the biological mechanisms of PRP, the various preparation techniques, and their applications in compound fracture care, this review aims to understand the current state of knowledge in the field thoroughly. Additionally, this review seeks to critically analyse the existing clinical evidence, present case studies and experiences, and discuss the ethical and regulatory considerations associated with using PRP in compound fracture management. Ultimately, this review aspires to offer valuable insights for clinicians, researchers, and healthcare professionals involved in fracture care, guiding them in making informed decisions about integrating PRP into treatment protocols. By examining the potential benefits, limitations, and future directions of PRP therapy in compound fractures, this review aims to contribute to the ongoing discourse on advancing regenerative approaches in orthopaedic medicine.

## Review

PRP preparation techniques

Centrifugation Methods

One of the critical aspects influencing the efficacy of PRP is the method of preparation, particularly the centrifugation process. Centrifugation serves as the cornerstone for isolating and concentrating platelets from whole blood. Various centrifugation methods have been employed, each with its unique set of parameters and considerations. Commonly utilised methods include differential centrifugation, density gradient centrifugation, and buffy coat [5]. Differential centrifugation involves the separation of blood components based on their varying densities. This method results in distinct layers, with the platelet-rich layer typically located between the red and white blood cell layers. Density gradient centrifugation employs a gradient medium to refine the separation process further, yielding a more concentrated and purer PRP product. The buffy coat method isolates the layer rich in platelets and leukocytes for subsequent processing [1].

Concentration and Activation Protocols

Once the platelets are separated, concentration protocols play a crucial role in determining the final therapeutic efficacy of PRP. Concentration can be adjusted based on the specific requirements of the clinical application. Some protocols involve a single spin to concentrate platelets. In contrast, others may employ a double-spin technique for further refinement; the choice of anticoagulant, centrifugation speed, and duration influence platelet concentration [6]. Activation of PRP is another essential step, as it transforms the platelets into their active form, releasing growth factors and other bioactive molecules. Common activation agents include calcium chloride, thrombin, or mechanical methods such as agitation. The activation protocol choice depends on the intended application, considering the desired release kinetics of growth factors and the overall therapeutic goals [7].

Quality Control Measures

Ensuring the reproducibility and consistency of PRP preparations is paramount for its clinical success. Quality control measures are integral to guarantee the safety and efficacy of the PRP product. Parameters such as platelet concentration, leukocyte content, and the presence of red blood cells must be rigorously assessed. Standardisation of preparation techniques, adherence to established guidelines, and the use of validated laboratory equipment contribute to the reliability of PRP preparations [8]. Moreover, quality control extends to the screening of donors, handling and storing blood components, and preventing contamination during the preparation process. Comprehensive documentation of the preparation process, including details on equipment calibration, reagent lot numbers, and any deviations from the standard protocol, ensures traceability and facilitates post-processing analysis [9].

Applications of PRP in compound fracture care

Acceleration of Bone Healing

PRP plays a fundamental role in accelerating bone healing within the context of compound fractures. This therapeutic application relies on the rich array of growth factors contained in PRP, including platelet-derived growth factor (PDGF), transforming growth factor-beta (TGF-β), and insulin-like growth factor (IGF). These growth factors stimulate critical cellular players, namely, osteoblasts and chondrocytes [10]. Osteoblasts are responsible for bone formation, while chondrocytes contribute to cartilage generation. Both cell types are indispensable in the intricate processes of bone regeneration. By concentrating these essential growth factors directly at the fracture site, PRP aims to amplify and optimise the biological mechanisms governing bone healing [10]. This targeted approach can potentially expedite the overall healing timeline, fostering a more robust and efficient regeneration of bone tissue. The ultimate objective is to reduce healing duration and enhance the healed bone's structural integrity, improving functional outcomes in individuals with compound fractures [10].

Enhancement of Soft Tissue Repair

Beyond its influence on bone healing, PRP showcases significant efficacy in augmenting the repair of soft tissues, a pivotal dimension in managing compound fractures. The traumatic nature of compound fractures often results in compromised soft tissues, encompassing muscles, tendons, and ligaments. PRP's capacity to stimulate fibroblasts, specialised cells crucial for tissue repair, and facilitate collagen synthesis emerges as a valuable asset in fostering the regeneration of damaged soft tissues [11]. This dual functionality, addressing both bone and soft tissue elements, positions PRP as a holistic and comprehensive therapeutic strategy for navigating the complex challenges of compound fractures. By promoting healing in both skeletal and surrounding soft tissue components, PRP holds promise in contributing to a more comprehensive and practical approach to managing complex fractures, potentially improving overall patient outcomes [11].

Reduction of Inflammation and Infection Risk

The inherent risk of infection and inflammation in compound fractures stemming from the exposure of the fracture site to the external environment underscores the critical need for effective interventions. PRP is a valuable therapeutic tool in this context, demonstrating inherent anti-inflammatory properties. Through modulation of the immune response, PRP effectively mitigates the inflammatory cascade typically associated with tissue injury in compound fractures. Additionally, the presence of antimicrobial peptides within PRP contributes significantly to diminishing the risk of infections at the fracture site, a particularly relevant consideration given the compromised nature of the injury [12]. By fostering an environment that not only is conducive to healing but also actively mitigates inflammation and infection risks, PRP holds the potential to minimise complications associated with these factors. This capacity to create a favourable microenvironment contributes to the overall optimisation of fracture repair, offering a promising avenue for addressing the complex challenges posed by compound fractures [12].

Pain Management in Fracture Recovery

Effective pain management is integral to the overall success of fracture recovery, influencing patient comfort, adherence to rehabilitation protocols, and overall satisfaction with the treatment journey. In this context, PRP stands out for its analgesic properties, offering a potential adjunct to pain management strategies for compound fractures. These analgesic effects can be attributed to PRP's ability to modulate pain receptors and reduce inflammation, two crucial components in the complex experience of pain [13]. By not only addressing the nociceptive pathways responsible for pain perception but also mitigating the inflammatory response, PRP has the potential to contribute significantly to a more comfortable and expedited recovery process for individuals grappling with the challenges of compound fractures. This dual action positions PRP as a promising addition to the multifaceted approach required for effective pain management in fracture recovery [13].

Clinical evidence and studies

Review of Published Studies on PRP in Compound Fracture Cases

PRP has emerged as a compelling area of investigation for treating bone fractures, with a particular focus on its potential impact on bone healing. A comprehensive systematic review encompassing preclinical and clinical studies has provided insights into the current evidence surrounding PRP's efficacy in fracture care. Notably, the findings indicate that while PRP demonstrated a notable reduction in the duration of bony healing, there was no discernible improvement in the healing rate of closed fractures [14]. Most published studies underscored the acceleration of fracture healing with PRP; however, it is crucial to note that the evidence was primarily categorised as level IV. This emphasises the need for further research to comprehensively understand PRP's efficacy and safety in the broader spectrum of fracture care [15,16].

Specifically, certain studies have presented statistically significant effectiveness of PRP, mainly when applied to fractures of the lower extremities [17]. Nonetheless, the overall effect of PRP on bone fracture treatment remains controversial and is under discussion within the scientific community [14]. To bridge these gaps in knowledge and resolve conflicting evidence, there is a pressing need for additional research, specifically prospective randomised clinical trials. These trials can provide a more robust and standardised assessment of PRP's effectiveness, shedding light on its potential role in managing compound fractures. Until then, the status of PRP in fracture care remains nuanced, emphasising the imperative for further rigorous investigation to establish its efficacy in diverse fracture scenarios [14].

Meta-Analyses and Systematic Reviews

PRP has garnered substantial attention in bone fracture treatment. A systematic review incorporating current evidence from preclinical and clinical studies revealed that PRP exhibited a capacity to shorten the duration of bony healing. However, it did not significantly improve the healing rate of closed fractures [18]. While most published studies reported the acceleration of fracture healing with the application of PRP, it's noteworthy that this evidence was primarily classified as level IV. Consequently, a call for further research is imperative to comprehensively understand PRP's efficacy and safety profile in the broader spectrum of fracture care [19,20].

Specifically, certain studies have highlighted PRP's statistically significant effectiveness, particularly in treating lower extremity fractures [21]. However, the overall impact of PRP on bone fracture treatment remains contentious and subject to ongoing debate within the scientific community [18]. To address these uncertainties, additional research, including prospective randomised clinical trials, is deemed essential, particularly to discern the effectiveness of PRP in cases involving compound fractures. A meta-analysis suggested the potential benefits of PRP as an adjuvant therapy for fracture patients, with a notable emphasis on mandibular fractures. Nevertheless, the meta-analysis underscores the necessity for further randomised controlled trials that utilise standardised outcomes to offer more conclusive insights into the role of PRP in fracture management [18]. This nuanced landscape emphasises the ongoing evolution of our understanding of PRP's potential in fracture treatment. It urges the scientific community to conduct rigorous research for more definitive conclusions and insights into its clinical applications.

Limitations and Gaps in Current Research

The clinical evidence surrounding the use of PRP in treating bone fractures presents a mixed landscape, underscoring several limitations and gaps in current research. Although specific studies have reported a potential benefit of PRP in shortening the duration of bony healing and accelerating the overall fracture healing process, it's crucial to note that most of this evidence falls within the level IV classification. This classification highlights the need for additional research, specifically prospective randomised clinical trials, to understand PRP's efficacy and safety in fracture care comprehensively [14,22,23].

Moreover, the overall impact of PRP on bone fracture treatment remains controversial within the scientific community, indicating diverse perspectives and interpretations of the available data [20]. While a meta-analysis has suggested potential benefits of PRP as an adjuvant therapy, particularly in the case of mandibular fractures, it is emphasised that further randomised controlled trials utilising standardised outcomes are necessary for more conclusive and robust findings [24].

In light of these considerations, it is apparent that the existing evidence regarding the effectiveness of PRP in bone fracture treatment has limitations. The need for more high-quality research is evident to address these limitations and provide a clearer understanding of the role of PRP in optimising outcomes for individuals with fractures. The ongoing dialogue and pursuit of rigorous research in this field are critical for advancing our knowledge and refining clinical practices related to PRP in bone fracture treatment.

Case studies and clinical experiences

Presentation of Notable Cases

The existing body of literature on using PRP in bone fracture treatment is characterised by a need for more published case studies, although those available highlight positive outcomes. One notable case report documented the application of PRP in a patient with a lateral femoral condylar stress fracture accompanied by osteonecrosis. Following PRP treatment, the patient experienced a substantial 85% improvement in pain and regained the ability to walk without a cane [25]. Another case report showcased the utilisation of PRP in a patient with a non-union fracture of the ulnar bone. After receiving PRP injections at the site of the failed union, the patient exhibited complete healing of the non-union, with the return of limb strength to normal levels [16].

While these case reports offer promising insights into the potential benefits of PRP in bone fracture treatment, it is essential to acknowledge the limited scope and the need for further research to evaluate its efficacy and safety in fracture care comprehensively. Most published studies on PRP and bone fractures predominantly fall under level IV evidence, indicating a lower level of certainty and emphasising the necessity for more rigorous research methodologies [10,14,15,25]. Specifically, the paucity of prospective randomised clinical trials in the current literature underscores the existing limitations. It highlights the crucial requirement for more high-quality research to ascertain the effectiveness of PRP in bone fracture treatment. As the field evolves, integrating robust evidence from well-designed studies will be pivotal in refining our understanding and enhancing the clinical applicability of PRP in fracture care.

Outcomes and Observations

PRP has emerged as a captivating focus in bone fracture treatment, yet the clinical evidence surrounding its efficacy presents a mixed picture. Some studies report positive outcomes in specific scenarios, notably in cases of non-union fractures. For instance, a randomised, double-blind, placebo-controlled clinical trial showcased significant advancements in the complete healing rate of long bone non-union fractures when treated with PRP, revealing an 81.1% complete healing rate compared to 55.3% in the placebo group [10]. However, the overall impact of PRP on bone fracture treatment remains a contentious subject, generating ongoing discussions within the scientific community [26].

A systematic review that comprehensively evaluated current evidence from preclinical and clinical studies uncovered that PRP displayed a capacity to reduce the duration of bony healing. However, it did not consistently improve the healing rate of closed fractures [20]. This underscores the need for further research, mainly through prospective randomised clinical trials, to determine PRP's effectiveness in bone fracture treatment conclusively [14,27]. While specific studies in the literature highlight positive outcomes, the overall evidence regarding the application of PRP in bone fracture treatment requires more definitive conclusions. A call for more high-quality research is evident to deepen our understanding of PRP's efficacy and safety in the nuanced landscape of fracture care. As such, the utilisation of PRP in bone fracture treatment stands as a promising but evolving area, demanding further scrutiny for a more comprehensive and conclusive assessment of its clinical potential.

Lessons Learned and Practical Considerations

Based on the available literature, PRP use in bone fracture treatment has shown promising results, but the overall evidence still needs to be conclusive. PRP has shown positive outcomes in some cases, such as non-union fractures and diabetic foot ulcers, by promoting healing and reducing wound size and depth [28]. However, more high-quality research is needed to confirm these findings. Most published studies on PRP and bone fractures are predominantly level IV evidence, and there is a lack of prospective randomised clinical trials [29,30]. Further research, including randomised controlled trials, is needed to determine the effectiveness of PRP in bone fracture treatment. PRP therapy increased complete wound closure or healing in lower extremity diabetic ulcers, shortened the time to complete wound closure, and reduced wound area and depth [28]. However, no significant changes were found regarding wound infection, amputation, wound recurrence, or hospitalisation [28]. The safety and efficacy of PRP in the Medicare population remain unclear, as Medicare-eligible older adults were underrepresented in the included studies [28]. Further research should focus on this population to determine the appropriate use of PRP in fracture care. While some positive outcomes are reported in the literature, the overall evidence regarding the use of PRP in bone fracture treatment is inconclusive, and more high-quality research is needed to fully understand its efficacy and safety in fracture care.

Comparative analysis with traditional treatments

Comparison with Standard Fracture Care Protocols

The use of PRP in managing non-union fractures has been a topic of interest in the literature. However, the evidence regarding the effectiveness of PRP in comparison to standard fracture care protocols still needs to be conclusive. Most published studies on using PRP for non-union fractures are predominantly level IV evidence, lacking prospective randomised clinical trials and level III evidence [15,27]. While some studies have reported that PRP accelerated fracture healing, the need for more randomised clinical trials is hampering PRP's successful clinical translation for non-union fractures [23]. A comprehensive overview of the use and effect of PRP in the initial treatment of fractures provided details of seven randomised clinical trials, one cohort study on the preparation and application of PRP, and the reported effect on radiographic healing and functional recovery. The follow-up of all studies was sufficient for reporting a reliable rate of fracture healing. However, the number of available and included studies could be higher, and future controlled studies with larger sample sizes and better-standardised use of PRP are needed to determine the actual effect of PRP in optimising patient care concerning bone healing [27]. While some studies have suggested the potential benefits of PRP in the treatment of non-union fractures, the current evidence is limited, and more high-quality research, including prospective randomised clinical trials, is needed to determine the effectiveness of PRP compared to standard fracture care protocols.

Advantages and Disadvantages of PRP in Comparison

PRP therapy offers several advantages and disadvantages compared to traditional treatments for various conditions, including bone fractures. Table 1 describes the advantages and disadvantages of PRP.

**Table 1:** Advantages and disadvantages of PRP PRP: platelet-rich plasma

Advantages of PRP	Disadvantages of PRP
PRP is a safe treatment using the patient's blood, reducing the risk of rejection and infection [31].	PRP injections lack standardised protocols, leading to diverse treatment outcomes [32].
PRP is a non-surgical, minimally invasive procedure applicable to various conditions such as ankle sprains, tennis elbow, knee sprains, osteoarthritis, rotator cuff injury, and more [31].	PRP is a supplemental treatment, potentially requiring additional therapies for long-term relief [32].
PRP injections facilitate swift healing and tissue regeneration, allowing patients a quicker return to daily activities compared to some traditional treatments [32].	The effectiveness of PRP injections is debated, and there are mixed results in the available literature [32].
PRP is a versatile treatment for sports trauma injuries, chronic tendonitis, osteoarthritis, skin rejuvenation, hair loss, and post-surgical healing [32].	-

PRP therapy offers several advantages: safety, non-surgical nature, rapid healing, and versatility. However, there are also some disadvantages, such as variability in treatment results, the supplemental nature of the treatment, and the limited evidence regarding its effectiveness. Further research and standardisation of protocols are needed to fully understand the benefits and limitations of PRP therapy compared to traditional treatments for bone fractures.

Integration of PRP with Conventional Therapies

PRP therapy can be integrated with conventional treatments to enhance healing. PRP is a safe and natural treatment that can be used alone or with other therapies. It has been effective in various medical fields, including orthopaedics, aesthetics, dentistry, and regenerative medicine [24,33]. When integrated with conventional treatments, PRP has the advantage of immediate preparation, safety, and minimal immune response, as it uses the patient's cells without further modifications [33]. However, the effectiveness of PRP in bone fracture treatment is still a matter of debate, and the evidence needs to be more conclusive. Some studies have reported that PRP can shorten the bony healing duration, but it may not necessarily improve the healing rate of closed fractures [33]. Therefore, while PRP can be integrated with conventional treatments, further research and standardised protocols are needed to determine its effectiveness in optimising patient care concerning bone healing [14,33].

Future directions and research recommendations

Emerging Technologies and Innovations in PRP

As PRP continues to gain recognition for its therapeutic potential in compound fracture care, there is a need to explore emerging technologies and innovations that could further enhance its efficacy. Advances in PRP preparation techniques, such as microfluidic devices and automated systems, offer the potential for standardisation and precision in the concentration of platelets and growth factors. Additionally, incorporating bioengineering strategies, such as developing PRP-loaded scaffolds or matrices, holds promise in providing a controlled and sustained release of bioactive factors at the fracture site. Investigating these emerging technologies will contribute to refining PRP applications and optimising their therapeutic benefits [20].

Areas for Further Investigation

While existing studies provide valuable insights into the applications of PRP in compound fracture care, several areas warrant further investigation to advance our understanding and improve clinical outcomes. Long-term follow-up studies are crucial to assess the durability and stability of the healing achieved with PRP therapy [19]. Comparative effectiveness research comparing PRP preparation methods, concentrations, and application timings will help establish standardised protocols for optimal outcomes. Furthermore, exploring the impact of patient-specific factors, such as age, comorbidities, and the nature of the fracture, on PRP efficacy will contribute to tailoring treatment approaches. Investigations into the cost-effectiveness of PRP compared to traditional fracture care methods will also be essential for its widespread adoption [34].

Potential for Personalised Medicine in PRP Application

The evolving field of personalised medicine opens up new possibilities for tailoring PRP therapy to individual patient characteristics. Genetic and molecular profiling could be employed to identify specific patient biomarkers that predict responsiveness to PRP treatment. Understanding how variations in patient factors influence the regenerative response to PRP will allow for more targeted and personalised interventions. Additionally, integrating imaging modalities, such as advanced radiographic and MRI techniques, could aid in the precise localisation of PRP delivery, ensuring optimal coverage of the fracture site. Investigating the potential for personalised medicine in PRP application is crucial for refining treatment strategies and maximising therapeutic outcomes for diverse patient populations [35].

Ethical and regulatory considerations

Informed Consent and Patient Communication

The ethical use of PRP in compound fracture care demands a robust framework for informed consent and transparent patient communication. Informed consent is a cornerstone of ethical medical practice, and patients undergoing PRP therapy for compound fractures should be provided with comprehensive information regarding the procedure, potential risks, benefits, and alternatives [35]. Ensuring that patients clearly understand the experimental or off-label nature of PRP in specific contexts is essential. Additionally, healthcare professionals must communicate the variable nature of outcomes and the evolving nature of PRP research. Open and honest discussions between healthcare providers and patients are crucial to fostering trust and respecting the autonomy of individuals seeking PRP as part of their fracture care [35].

Regulatory Framework for PRP Use in Fracture Care

The regulatory landscape surrounding the use of PRP in fracture care is evolving, and establishing a clear framework is essential to ensure patient safety and ethical practice. Regulatory authorities should collaborate with healthcare professionals and researchers to develop guidelines that address the unique considerations of PRP therapy. This includes defining standardised protocols for PRP preparation, ensuring the quality and safety of PRP products, and establishing criteria for the ethical use of PRP in compound fracture cases. A practical regulatory framework will safeguard patient welfare and provide a foundation for responsibly advancing research and clinical applications [35].

Addressing Ethical Concerns and Patient Safety

The ethical use of PRP in compound fracture care necessitates a vigilant approach to addressing ethical concerns and ensuring patient safety throughout the treatment process. Continuous monitoring of adverse events and long-term outcomes is essential to identifying and mitigating potential risks associated with PRP therapy. Ethical considerations also extend to issues such as equitable access to PRP treatment, avoiding unnecessary financial burdens on patients, and preventing the inappropriate marketing or promotion of PRP as a panacea for compound fractures. Healthcare professionals and researchers must prioritise patient safety, adhere to ethical guidelines, and engage in ongoing dialogue to address emerging ethical concerns as PRP therapy advances [20].

## Conclusions

Examining PRP in the context of compound fracture care reveals a promising and multifaceted approach to orthopaedic regenerative medicine. The review has illuminated key findings, emphasising the potential for PRP to accelerate bone healing, enhance soft tissue repair, reduce inflammation and infection risks, and contribute to effective pain management in fracture recovery. The implications of incorporating PRP into compound fracture care protocols suggest a transformative shift in the standard of orthopaedic treatment. As emerging technologies and personalised medicine strategies evolve, the future promises a more tailored and effective use of PRP, potentially revolutionising patient outcomes and overall quality of life. However, amidst optimism, navigating ethical considerations, refining treatment protocols, and ensuring responsible and transparent communication with patients are crucial. In harnessing the healing power of PRP, the collaborative efforts of healthcare professionals, researchers, and regulatory bodies will play a pivotal role in realising the full potential of this regenerative therapy and reshaping the landscape of orthopaedic care for individuals with compound fractures.
